# Rapatar, a nanoformulation of rapamycin, decreases chemically-induced benign prostate hyperplasia in rats

**DOI:** 10.18632/oncotarget.3929

**Published:** 2015-04-26

**Authors:** Ekaterina A. Lesovaya, Kirill I. Kirsanov, Elena E. Antoshina, Lubov S. Trukhanova, Tatiana G. Gorkova, Elena V. Shipaeva, Ramiz M. Salimov, Gennady A. Belitsky, Mikhail V. Blagosklonny, Marianna G. Yakubovskaya, Olga B. Chernova

**Affiliations:** ^1^ Department of Chemical Carcinogenesis, Blokhin Cancer Research Center, Moscow, Russia; ^2^ Tartis-Aging LLC, Moscow, Russia; ^3^ Department of Cell Stress Biology, Roswell Park Cancer Institute, Buffalo, NY, USA; ^4^ Everon Biosciences, Inc., Buffalo, NY, USA

**Keywords:** gerotarget, aging, benign prostatic hyperplasia, mTOR, rapamycin

## Abstract

Benign prostatic hyperplasia (BPH) is the most common age-related disease in men. Here we tested the efficacy of Rapatar, a micellar nanoformulation of rapamycin, in two rat models of BPH: testosterone-induced and sulpiride-induced hyperplasia in ventral lobes and lateral/dorsal lobes, respectively. We found that Rapatar prevented hypertrophic and hyperplastic abnormalities and degenerative alterations in both BPH models. Rapatar normalized weight of the lateral lobes in sulpiride-induced BPH, the most relevant animal model of human BPH. Unlike Finasteride, a standard therapy of BPH, Rapatar reduced inflammation caused by sulpiride. No obvious side effects of Rapatar were detected. Our data provide a rationale for clinical trials of Rapatar in patients suffering from BPH.

## INTRODUCTION

Benign prostatic hyperplasia (BPH) is the most frequent disease in men. This pathology affects 50% of men over the age 50 and 90% of men over the age 80. BPH is associated with increased incidence of prostate cancer [1].

Current treatments for BPH are based on three main strategies: inhibition of 5-α-reductase (5αR), attenuation of gonadotropin-releasing hormone, and blocking of α-adrenoreceptors. Inhibitors of 5-α-reductase (Finasteride) suppress conversion of testosterone into a more potent metabolite, 5α-dihydrotestosterone (DHT). The second strategy depends on suppression of gonadotrophin secretion, which otherwise stimulates testosterone production. Lastly, alpha-1-blockers inhibit the effect of noradrenaline on prostate smooth muscle cells, which represent more than 60% of the enlarged gland, thereby reducing prostate tone and bladder outlet obstruction [2-4] However, these medications improve lower urinary tract symptoms by less than 35-50% and cause side effects.

BPH, which affects elderly men, is a clear cut age-related disease [5, 6]. mTOR is involved in cellular senescence [7]. Rapamycin inhibits cellular senescence [7] and slows down organismal aging. Rapamycin also prevents cancer in rodents and humans [8-10]. Rapamycin decreases cellular hypertrophy [7]. These activities of rapamycin may be useful for treatment of BPH. Recently, Rapatar, water-soluble nanoformulated micelles of rapamycin, was shown to delay cancer in cancer-prone mice [11].

There are two widely used methods of BPH induction. Testosterone and prolactin stimulate development of BPH [12]. Animal models of BPH are based on prostate overstimulation by these hormones [13, 14]. In testosterone-induced model, administration of testosterone causes hyperplasia in ventral lobes of the rat prostate, analogous to morphological changes in human BPH [15-17]. In sulpiride-induced model, sulpiride stimulates prolactin production by the pituitary gland, thus causing hyperplasia in the lateral and dorsal prostate lobes. Levels of serum prolactin are increased with age. Therefore, the sulpiride model is closely related to BPH in humans [18]. Here we compared therapeutic effects of 5αR inhibitor Finasteride, commonly used in BPH therapy, and Rapatar in two rat models of BPH.

## RESULTS

### Rapatar prevents hypertrophy in sulpiride-induced BPH

In testosterone group, testosterone-filled silastic tubes were implanted subcutaneously (Table 1). In sulpiride group, hyperprolactinemia was induced by daily intraperitoneal (i.p.) injections of sulpiride. On day 5, animals were given either Rapatar or Finasteride or left untreated (Table 1).

**Table 1:** Treatment schedules

Group*	Description	BPH induction	+ Gavage (three times a week)
1	T-Control	Tubing without Т	
2	T-BPH	Testosterone model (T) Subcutaneously implanted with Silastic tubes containing 40 mg of Testosterone	0.2% HPMC
3	T-BPH +R 0.5		Rapatar 0.5 mg/kg
4	T-BPH +R 1.5		Rapatar 1.5 mg/kg
5	T-BPH +R 3.0		Rapatar 3.0 mg/kg
6	T-BPH +F 10		Finasteride 10 mg/kg
7	S-Control	Daily intraperitoneal injections of physiological saline	
8	S-BPH	Sulpiride model (S) Daily intraperitoneal injections of 30 mg/kg Sulpiride	0.2% HPMC
9	S-BPH +R 0.5		Rapatar 0.5 mg/kg
10	S-BPH +R 1.5		Rapatar 1.5 mg/kg
11	S-BPH +R 3.0		Rapatar 3.0 mg/kg
12	S-BPH +F 10		Finasteride 10 mg/kg

* Groups 1 and 2 contained 9 rats; groups 3 to 12 contained 10 rats

**Figure 1:**
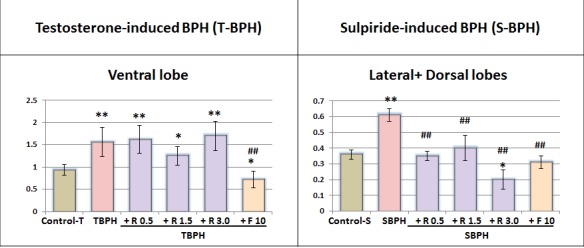
Relative weight of the ventral lobe and or lateral+dorsal lobes of the prostate Lobes weight (mg) divided by total body weight (g). Values are means ± SE; Control +T and TBPH groups *n* = 9, other groups *n* = 10. Animals were treated as described in Table 1. Superscript signs show significant difference: * – from Control-T or Control-S, ** - *p* < 0.01, *p < 0.05, # – from TBPH or SBPH, ## - *p* < 0.01, # - *p* < 0.05. R – Rapatar; F- Finasteride.

Testosterone induced a 1.6-fold increase in the ventral lobe weight (Figure 1A). Finasteride but not Rapatar prevented weight gain of prostate lobes. Yet, as shown in our study (below), Rapatar prevented hyperplastic and degenerative histological abnormalities and inflammation caused by testosterone.

In sulpiride-induced model, we analyzed the weight of lateral and dorsal lobes because these lobes are enlarged by sulpiride [14], [19]. Sulpiride increased weight of lateral and dorsal lobes (Figure 1B). At all 3 doses used, Rapatar prevented the effect of sulpiride. At dose 3.0 mg/kg, the weight of the lobes was even lower than the weight in control group (Figure 1B).

### Rapatar normalized prostate gland structure

**Figure 2:**
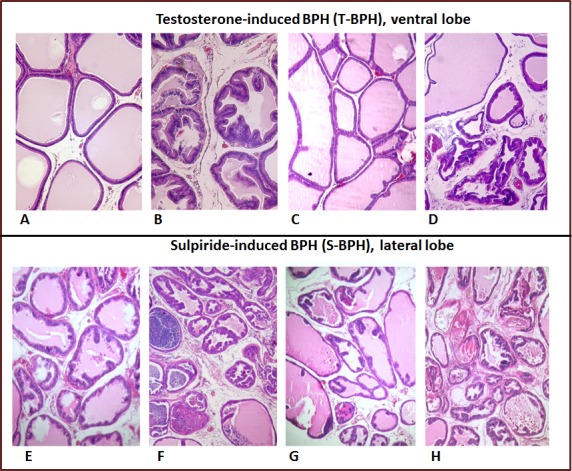
Histology of ventral (A-D) and lateral prostate (E-H) **A.**Venstral lobe of intact rats, H&E, x100; **B.** Testosterone-induced BPH (irregular acinar shape with villous projections of different sizes into the lumen), x80. **C.** Rapatar normalized GP ventral lobe structure, H&E, x60; **D.** 30 days of co-administration of Testosterone with Finasteride (glands are partially atrophic, with dilated, angular profiles, adenomatous hyperplasia), H&E, x60. **E.** Prostatic lateral lobe of intact rats (Control-S), H&E, x60; **F.** 30 days after Sulpiride treatment (adenomatous hyperplasia manifested by acinar epithelium proliferation, inflammatory infiltration of the stroma), H&E, x60. **G.** Rapatar normalized LP structure, H&E, x60; **H.** 30 days of co-administration of Finasteride with Sulpiride (glands are partially atrophic, with dilated, angular profiles,adenomatous hyperplasia, inflammatory infiltration of the stroma), H&E, x60.

**Figure 3:**
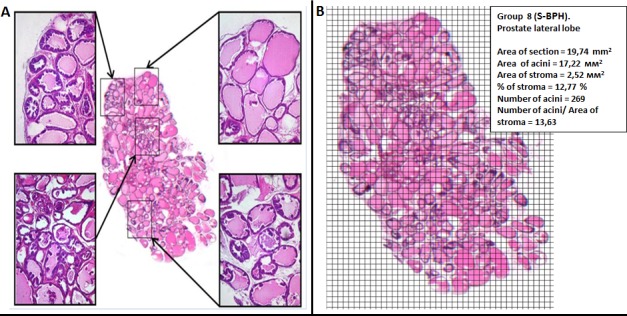
Histomorphometric analysis in PG **A.** Prostate structure in different section areas. **B.** A number of morphological changes within the total section of prostate lobe.

The rat prostate gland has tubuloacinar structure (Figure 2A, 2E). The acini are lined with epithelium and surrounded by loose, fibrous connective tissue containing smooth-muscle fibers and blood vessels. The acini located along the periphery of the lobe are smaller, but their walls are more folded. The epithelial cells of the acinar lining are cylindrical, prismatic or cuboidal with a basal nucleus. The dorsal and lateral lobes are located tightly against each other to the extent that they appear macroscopically as a single lobe. But a very thin connective-tissue septum can be used to distinguish between them microscopically. The acini of the lateral and dorsal lobes are smaller than those of the ventral lobes and have a more pronounced degree of folding (Figure 2A, 2E). As small acini areas are located in the gland section irregularly, the stroma and small acini percentage assessment may depends on the occasional or subjective investigator choice of the sites (Figure 3A). To prevent the bias, we analyzed entire lobe sections scanned in full scale and overlaid with a grid in order to estimate the relative stroma and acini areas (Figure 3B). We did not reveal any change in the relative stroma areas in bothTestosterone-induced (Figure 2A, 2B) and sulpiride-induced BPH (Figure 2E, 2F, Table 2). In both testosterone- and in Sulpiride-induced BPH, adenomatous hyperplasia was manifested by an increased proportion of small acini surrounding large glands (Figure 2B, 2F, Table 2), in agreement with previous results [17]. In sulpiride-induced BPH, proportion of small acini was 40±2.4%, compared with 21±1%, in control. In Testosterone-induced BPH, there was 1.75 fold increase of small acini portion in the ventral lobes: from 21.2±2.9% to 37.2±2.1% (Table 2).

**Table 2:** Relative area of small acini in prostate gland (PG)

Groups	% of stroma	Number of acini/area of stroma	Relative quantity of small acini (%) in section	Ratio experiment/control1)
BPH induced by Testosterone in ventral lobes				
T-Control	22.41±1.62	9.18±0.48	21.2±2.9	
T-BPH	23.49±3.73	7.40±0.58	37.2±2.1	1.75
T-BPH +R 0.5	20.99±2.79	7.16±0.41	16.5±1.0	0.8
T-BPH +R 1.5	23.88±2.84	7.29±0.39	18.5±1.5	0.9
T-BPH +R 3.0	23.04±1.6	6.54±0.40	26.5±3.0	1.25
T-BPH +F 10	20.21±2.17	10.38±0.69	40.8±2.0	1.9
B-PH induced by Sulpiride in lateral and dorsal lobes				
S-Control	13.8 ±1.9	11.9 ±0.8	21±1	
S-BPH	12.5 ±1.8	9.9 ±0.73	40±2.4	1.9
S-BPH +R 0.5	10.6 ±1.2	10.9 ±0.7	15.1±1.5	0.7
S-BPH +R 1.5	9.7 ±1.2	14.6 ±1.7*	18.6±2.2	0.9
S-BPH +R 3.0	10.9 ±2.0	16.9 ±1.83*#	18.4±2.3	0.9
S-BPH +F 10	14.7 ±1.5	12.2 ±1.03	35.9±2.4	1.7

Ratio of small acini (<0.06мм2) per cent in lobes of rats treated by Testosterone (T) or Sulpiride (S) in combination with Rapatar (R) or Finasteride (F) to that induced by Testosterone or Sulpiride only.

Significantly different: * – from Control-T or Control-S

* p < 0.05;

# – from T-BPH or S-BPH

# p < 0.05

In sulpiride-induced BPH, Rapatar caused normalization of prostate gland structure (Figure 2G). Percent of small acini in the lateral lobes decreased to 15.1±1.5%, 18.6±2.2% and 18.4±2.3% at doses of 0.5, 1.5 or 3 mg/kg (versus 40.6±2.4% for Sulpiride alone and 21±1% in control group) (Table 2). Similar, in testosterone-induced BPH, Rapatar caused normalization of gland structures (Figure 2C, Table 2). In contrast, Finasteride did not decrease a number of small acini in either sulpiride- or testosterone-induced BPH (Figure 2D, 2H, Table 2).

### Rapatar reduced papillary projections and hyperplasia in testosterone-induced BPH

**Figure 4:**
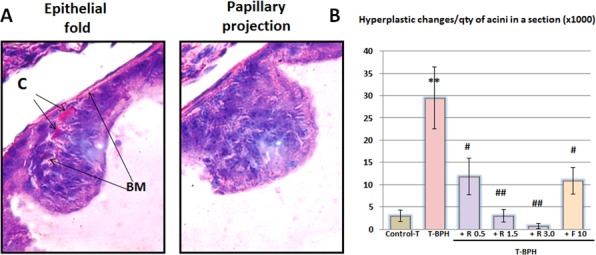
Histological hyperplastic changes in the prostate C-capillar, BM- basal membrane. Data are presented as a mean value ± standart error (* – statistically significant difference from Control-T, ** -*P* < 0.01, ** -*P* < 0.05, # - statistically significant difference from TBPH, ## -*P* < 0.01, # -*P* < 0.05).

Hyperplasia of the acinar wall epithelium induced by testosterone in ventral lobes was manifested not only by adenomatous hyperplasia, but also by proliferation of the epithelial cells in the form of papillary projections, protruding into the lumina of the acini (focal hyperplasia) and by the appearance of multilayered epithelium areas of varying length along the acinar wall (diffuse hyperplasia or hyperplastic nodule) [13, 15]. Papillary projections could be distinguished from acinar wall folds, which often occur naturally, as the basal membrane also forms a fold within the fold of the acinar wall (Figure 4A). In contrast, there is no basal membrane in the papillary projections (Figure 4B). The number of papillary projections and amount of diffuse hyperplasia were estimated on the same section on which the proportion of small acini was determined. A ten-fold rise in the average number of focal proliferation (both of papillary and diffuse) was observed in the ventral lobes in Testosterone-BPH). Rapatar at doses 0.5, 1.5, and 3.0 mg/kg (TBPH + R 0.5; TBPH + R 1.5; TBPH + R 3.0), reduced this focal proliferation 2.5-, 9.9- and 42.1-fold, respectively.

### Rapatar prevents inflammation in prostate glands in sulpiride-induced BPH

**Figure 5:**
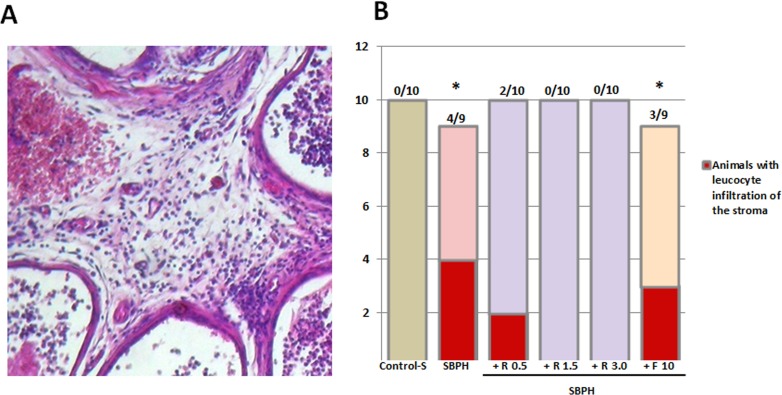
Sulpiride-induced inflammation in PG lateral lobes **A.** Stromal and periglandular inflammation induced by Sulpiride (S-BPH): lymphocytes in the stroma and in the intraepithelial space. **B.** Effect of Rapatar or Finasteride on prostate inflammation induced by Sulpiride (* – statistical difference from Control-S, *p* < 0,05)

There was no leukocyte infiltration of the stroma and acini in control rats. Sulpiride induced leukocyte infiltration in prostate gland tissue (Figure 5A). Signs of non-bacterial prostatitis at the lateral lobes were observed in 4 of 10 animals of this group (Figure 5B). Stromal leukocyte infiltration was also found in 33% of the animals after exposure to Finasteride. Leukocyte infiltration of the stroma was observed only in 2 out of 10 animals treated with Rapatar at 0.5mg/kg dose. Inflammation was not found in any rats treated with Rapatar at doses of 1.5 and 3.0 mg/kg (Figure 5C).

### Rapamycin decreases body weight gain in S-BPH

**Figure 6:**
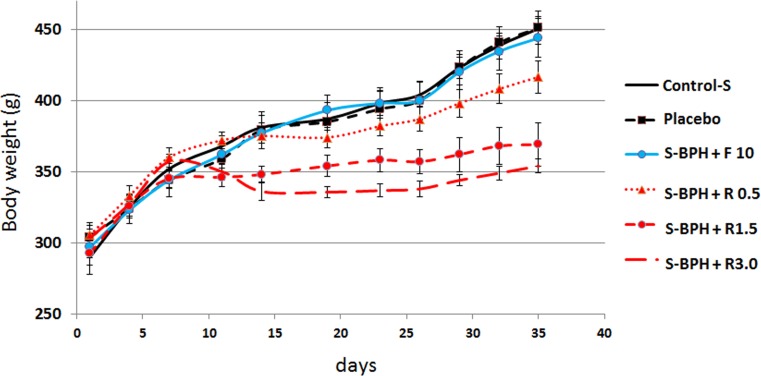
Effect of Rapatar on body weight S-BPH: Sulpiride-induced BPH (Groups #7– 12). Body weight was measured twice a week.

During the experiment, rats gained significant weight. This weight gain is explained by housing of each rat in individual cage that reduces stress and is commonly associated with weight gain. The same weight gain was observed in control BPH groups and in BPH groups treated with Finasteride (Figure 6). In S-BPH, Rapatar partially prevented weight gain. This effect was dose dependent (Figure 6). At doses 1.5 mg/kg and 3 mg/kg, Rapatar decreased weight by 13% and 17, respectively (Figure 6). It was previously reported that rapamycin decreased obesity in rodents on high-fat diet [20-23].

## DISCUSSION

Here we showed that, in a dose-dependent manner, Rapatar (a new oral formulation of rapamycin) prevented sulpiride-induced BPH. Sulpiride induced cystic enlarged acini, with atrophic and degenerative changes, loss of secretion and destruction of the epithelial lining. In this model, Rapatar normalized both the weight of prostate lobes and their tissue architecture. Rapatar decreased the cystic transformation of acini caused by sulpiride. Most of the acini were lined with secretory epithelium. In the surrounding area, a fewer number of small acini were observed. Thus, Rapatar abrogated prostate enlargement.

Rapatar also prevented pathological histological alterations caused by testosterone, while minimally affecting the lobes weight.

Effects of Finasteride on gland tissue in Sulpiride-induced and testosterone-induced BPH were similar. Prostate glands contained large empty acini with degenerating epithelium, surrounding by foci of small acinar proliferation. So although Finasteride decreased weight of the prostate lobes, this decrease was in part due to degeneration. In contrast, Rapatar prevented degeneration and normalized the tissue structure.

Noteworthy, the expression of androgen receptors depends mainly on testosterone in the dorsal/ventral lobes and on prolactin in lateral lobes [24], [25]. Therefore, both stimulating effects of Sulpiride and inhibitory (therapeutic) effects of Rapatar were mainly evident in lateral lobes.

We emphasize that sulpiride-induced BPH is a physiological model related to human BPH. First, levels of prolactin are increased in men with aging. The sulpiride model reproduces the hormonal background on which BPH develops in aged men. Second, lateral lobes are mainly involved in human BPH. Lateral PG lobes in rats and in men are most similar in their embryonic development, structure and function. Therefore, prevention of S-BPH by Rapatar in rats suggests that Rapatar will be most likely effective in BPH in men.

In men, BPH is accompanied by inflammation in the stroma and by advances to suppurative exudate in the acinar lumen. Similar alterations were observed in rats treated with testosterone and sulpiride, in agreement with previous reports [12, 14].

Rapatar decreased sulpiride-induced inflammation. In fact, rapamycin has anti-inflammatory activity [26- 28].

Finasteride reduced lobe weight due to loss of secreting epithelial cells in the acini, while Rapatar had a milder effect, inhibiting secretion of preserved secreting epithelium. Therefore, Rapatar and Finasteride could be combined for therapy of BPH. This needs additional study.

Finasteride can cause anti-androgen resistance, producing hormone-resistant tumors. In contrast, rapamycin prevents numerous types of cancer in rodents and humans [8-10]. In addition, rapamycin prolongs life span and prevents age-related diseases in mice [29-34].

Therefore, Rapatar has a number of advantages as a potential agent for prevention and treatment of BPH in men. Given that rapamycin is a clinically approved drug, clinical trials of Rapatar for treatment and prevention of BPH are warranted.

## MATERIALS AND METHODS

### Animals

Animal studies were performed in accordance with the guidelines for the Care and Use of Laboratory Animals of the Ethics Committee at the State-Funded Institution, N. N. Blokhin CRC, and done in accordance with the Geneva Convention of 1985 regarding international principles for biomedical research involving the use of animals, and with the 2000 revision of the Declaration of Helsinki concerning the humane treatment of animals.

Male Wistar rats (240-280 g) were kept in standard conditions with unrestricted access to food and drinking water. Rats were randomized, marked, weighed and housed individually. They were provided *ad libitum* with water and a standard laboratory chow. Body weight was measured twice a week at the same time (9:00–11:00 am).

### Experimental groups

Treatment schedules for experimental groups are shown in Table 1.

### Testosterone-induced BPH (T-BPH)

Groups 2-8: rats were implanted subcutaneously over the scapular region with testosterone-filled Silastic tubes containing 40 mg of testosterone as described [42]. In control group 1, the Silastic tubes were implanted without testosterone. On day 5 after tube implantation, rats (groups 3-8) were given either Rapatar (doses 0.5, 1.5 and 3.0 mg/kg, three times a week) or Finasteride (dose 10 mg/kg, three times a week). Both drugs were diluted in 0.2% HPMC and given by gavage for 30 days. Animals in groups 1 and 2 (T-Control and T-BPH) were gavaged with the solvent (Table 1).

### Sulpiride-induced BPH (S-BPH)

Hyperprolactinemia was induced by daily intraperitoneal injections of 30 mg/kg Sulpiride (diluted in 0.2% HPMC) for 28 days, as described [12]. On day 5 after the start of Sulpiride injections, rats (groups 7-12) were gavaged daily either with Rapatar (S-BPH +R 0.5; S-BPH +R 1.5; S-BPH +R 3.0) three times a week or by Finasteride (S-BPH +F10) for 30 days. Animals in groups 7 and 8 (S-Control and S-BPH) were gavaged with the solvent (Table 1).

### Necropsy and histology

Necropsy and removal of the prostate gland for histological evaluation were performed at day 35. The prostate lobes were dissected and weighed, and their volume was measured. The tissues were processed for light microscopy by fixing in 10% neutral buffered formalin, dehydrating, and embedding in paraffin. Histological analysis was performed on serial sections obtained from prostatic lobes stained by hematoxylin-eosin.

### Assessment of the stromal component and small acini portions in the whole PG section

Fifteen sections of the analyzed lobe of the prostate were examined microscopically and 1 section was selected as being the most representative for the animal. The chosen section was scanned in full scale and overlaid with a grid in order to estimate the area of the stroma on the entire examined section (Figure 3B).

The known area of each square (0.06 mm^2^) makes it possible to determine the area occupied by the entire section and separately by the stroma and to calculate the number of small acini (those measuring less than 0.06 mm^2^) in the section as a whole.

One of the methods for evaluation of hyperplastic changes is based on measurement of the surface occupied by acinar epithelium in a microscopic view field [19]. The estimation results may depend considerably on the subjective choice of the site by the investigator (Figure 3A). We determined the proportion of small acini within the area of the whole section using scanned in full scale section overlaid with a grid. At low magnification, the total number of acini is evaluated in the sections, as well as the number of small acini (less than 0.06 mm^2^ in size). The percentage of the small acini to the total number is calculated and this parameter corresponds closely to the area of the acinar epithelium and reflects the development of hyperplasia in an objective way.

### Hyperplastic changes of the acinar epithlelial lining

Hyperplasia of the acinar wall epithelium was manifested by proliferation of the epithelial cells forming papillary projections protruding into the acinar lumen (focal hyperplasia) and by the appearance of areas of multilayered epithelium of varying length along the acinar wall (diffuse hyperplasia).

The number of papillary projections and amount of diffuse acinar wall thickening were estimated on the same section from which the quantity of acini was calculated. When estimating the papillary projections, the latter were differentiated from normally occurring acinar wall folds in which the basal membrane exists (Figure 4). In contrast, there is no basal membrane in the papillary projection. Rather, the cells of the cuboidal epithelium located close to the basal membrane are smaller and more basophilic, forming sites of focal proliferation. In addition to the total number of papillary projections, their multiplicity was also determined. For this, the total number of papillary projections was divided by the number of acini in the section.

### Inflammation

In the case of inflammation, leukocyte infiltration of the stroma was observed and documented. An account was made of the number of animals in a group having this change.

### Statistical analysis

Prostate weight relative to the body weight. Significance was determined using Student's t-test. Significance in inflammation frequency in different groups was determined by the Neyman-Pearson approach.
